# Candidate Coatings for Lead Fast Reactor Components: A Systematic Screening of Liquid-Lead Compatibility—Part I

**DOI:** 10.3390/ma19183952

**Published:** 2026-09-17

**Authors:** Andrea Ventrella, Francesca Bussi, Daniele Cico, Francesca Ecclesia, Mattia Salvi, Chantal Vannini, Cyril Pudoyer, Davide Loiacono, Francisco García Ferré

**Affiliations:** 1 *new*cleo S.p.A., Via Giuseppe Galliano 27, 10129 Torino, TO, Italy; 2 *new*cleo S.p.A., C.R. Brasimone, Località Brasimone, 40032 Camugnano, BO, Italy; 3 *new*cleo S.A., 9 Rue des Cuirassiers, 69003 Lyon, France

**Keywords:** lead fast reactor, liquid lead corrosion, protective coatings, surface engineering, alumina-forming alloys, thermal spray, cold gas spray, chromium electrodeposition, lead penetration, coating degradation

## Abstract

**Highlights:**

- Conducted a systematic screening of metallic coatings to assess corrosion resistance in liquid-lead environments.
- Established a standardized qualification methodology for protective coatings in lead-cooled fast reactors (LFRs).
- Evaluated the protective efficacy of APS, HVOF, CGS, and chromium plating against substrate degradation.
- Identified coating integrity and defect connectivity as the primary drivers of liquid-lead ingress.
- Demonstrated that coating performance is fundamentally dictated by chemical composition and microstructure.

**Abstract:**

The development of protective coatings is a key requirement to extend the operating window of structural materials in lead-cooled fast reactors, particularly at temperatures where corrosion of conventional steels becomes increasingly challenging to control. In this work, a code-oriented, systematic and technology-agnostic screening of candidate coating systems was performed to assess their chemical compatibility with liquid lead under representative and controlled conditions. Different coating technologies and material systems were investigated. Part I of the work covers atmospheric plasma spraying, high-velocity oxy-fuel spraying, and cold gas spray of Al-rich austenitic and ferritic alloys, and chrome electrodeposition. The results show that, while Al-rich metallic coatings can effectively protect the underlying substrates under the investigated conditions, Cr-based materials studied here cannot. Such an outcome provides a comparative basis for the down-selection of coating technologies for LFR components.

## 1. Introduction

The use of lead or lead-bismuth as a coolant in fast reactors is appealing due to a number of intrinsic safety features, favourable neutronic properties and interesting thermal performance. Such appeal has encouraged a number of nations to work on the development of Lead Fast Reactors (LFRs) since the late 50’s. The former Soviet Union was the first to develop lead-bismuth cooled fast reactors, and operated LFR-powered submarines for a staggering 80 cumulative years during the Cold War. More recently, Russia, Japan, China and Europe have continued the work on LFR technology. Russia leads the charge with Rosatom’s BREST-OD-300 [1], while European players such as *new*cleo, the EAGLES consortium, and Blykalla are working on their LFR-AS-200 [2], EAGLES-300 [3] and SEALER [4] designs, respectively.

Over time, the operational experience [5] and the research conducted in Europe [6,7] have led the international LFR community to switch to pure lead as the most promising coolant. Compared to lead-bismuth, lead has a higher melting point, which brings along operational hurdles. However, the use of lead-bismuth entails a higher production of Po-210. This requires careful management of decay heat and strict radioprotection measures. Moreover, recent research points towards a higher susceptibility of steels to liquid metal embrittlement in lead-bismuth as compared to lead, especially ferritic steels [8,9,10]. By contrast, both coolants display corrosive effects on structural steels, and corrosion kinetics become hard to manage above 550 °C [6]. While the latest developments have demonstrated that corrosion can be effectively mitigated via oxygen control at operating temperatures in the range of 500 °C [7], an increase beyond 550 °C would unlock a desirable increase in the overall system efficiency (and thus economic return). Achieving such a goal is widely recognized as one of the most important technological challenges for LFRs. There are different approaches for achieving such goals. Russian designs rely mostly on Si-rich steels [11,12] for components and concrete for vessels, with a semi-integrated design [1]. Integrated pool designs operating at high temperature [4], such as *new*cleo’s or Blykalla’s USA designs, rely on FeCrAl bulk alloys [13,14,15,16], surface modifications [17], and weld-overlay of alumina-forming austenitic (AFA) steel [18,19]. The EAGLES consortium and *new*cleo rely on a phased approach for their projects in Europe, starting at low temperatures with oxygen control and corrosion allowance, followed by a second phase at high temperature where surface modifications and/or new materials are deployed [20].

The use of coatings has received great attention by the scientific community so far [21,22,23,24,25,26,27,28,29,30,31,32,33,34]. These investigations usually focus on a particular aspect (or a number of aspects) of a specific coating method and material and develop details (verticalize) on such aspects thereafter. The main focus has been on testing corrosion protection [34] and, occasionally, ion irradiation experiments to explore the potential effects that neutron bombardment would induce in an actual LFR [21,22,23,24,29,32]. The target application investigated has usually been fuel cladding protection; instead, little attention has been paid so far to identify which types of coatings would best suit other types of components based on geometry, size, and operational conditions. The literature on coatings for liquid-lead applications remains highly fragmented, as most studies investigate individual coating systems under specific and often non-comparable testing conditions. Differences in substrate material, exposure temperature, oxygen concentration, test duration, lead chemistry, and post-exposure characterization protocols make it difficult to directly assess the relative performance and transferability of different coating solutions. In this context, horizontal screening refers to the systematic comparison of multiple coating material–technology combinations under a common experimental framework. Rather than optimizing each system independently at this stage, the objective is to establish a consistent comparative baseline for early down-selection. The most promising candidates identified through this first screening can then be advanced to a subsequent phase involving mechanistic validation, process optimization, and application-specific qualification under more representative service conditions [35].

In this work, we investigate a broad range of coating technologies and coating materials within the framework of one of *new*cleo’s strategic materials R&D programs [20]. The screening approach adopted here is aligned with the code-oriented methodology to qualify coatings outlined by Pudoyer et al. [35], in which surface engineering is considered a key enabling route to improve the compatibility of structural materials with lead-based coolants.

The screening campaign encompasses up to one hundred combinations of coating materials and deposition technologies. The primary objective of this first phase is to assess, in a systematic and technology-agnostic manner, the chemical compatibility of different coated systems with liquid lead.

This paper represents the first contribution in a series of different publications dedicated to the evaluation of candidate coating solutions for lead fast reactor applications. The present work focuses on seven selected combinations of coating technology and coating material:

- Atmospheric plasma spraying (APS) of alumina-forming austenitic steel (AFA);
- Atmospheric plasma spraying (APS) of alumina-forming ferritic steel (AFF);
- High-velocity oxy-fuel spraying (HVOF) of alumina-forming austenitic steel (AFA);
- High-velocity oxy-fuel spraying (HVOF) of alumina-forming ferritic steel (AFF);
- Cold gas spraying (CGS) of alumina-forming austenitic steel (AFA);
- Cold gas spraying (CGS) of alumina-forming ferritic steel (AFF);
- Chromium electrodeposition.

The material–technology combinations investigated in this first horizontal screening were selected jointly, so that coating chemistry and deposition route could be evaluated together against the corrosion mechanisms relevant to liquid lead and the practical constraints of LFR component design, as will be discussed in the Discussion section.

Depending on a number of conditions, the degradation of structural steels in liquid lead can be driven by dissolution of alloying elements, selective leaching, liquid-metal penetration, or by the growth of protective oxide scales under controlled oxygen activity [6,7]. On the basis of the potential to form protective oxide layers, two classes of materials were selected as benchmarks for this study: alumina-forming systems (two types: AFA, AFF) (or more precisely, Al-rich systems with potential to form Al-rich oxide layers) and electrodeposited Cr (potentially chromia-forming). AFA and AFF-type alloys may promote growth of Al-rich oxide scales, generally more stable and slower-growing than Fe- or Cr-rich oxides at LFR-relevant temperatures [13,15,19]. AFA combines austenitic metallurgy with aluminum oxide -forming capability, while FeCrAl represents an AFF reference that performs well in lead and lead-bismuth eutectic [13,15,19]. Comparing AFA and FeCrAl under identical exposure conditions isolates whether protectiveness is controlled mainly by Al availability and oxide formation, or additionally by matrix chemistry and deposition-induced microstructure. Electrodeposited Cr was retained as a materially distinct benchmark: chromium coatings are industrially well established and generally regarded as effective corrosion barriers via chromia-scale formation [36,37,38] but in liquid lead their performance depends critically on coating continuity and defect population.

These materials were paired with four deposition routes chosen to span the dominant bonding mechanisms available for metallic coatings. APS and HVOF are mature, industrially established thermal spray processes, differing mainly in flame temperature and particle velocity: APS favors high deposition rates at the cost of increased particle oxidation and lamellar, splat-dominated microstructures, while HVOF’s lower flame temperature and higher kinetic energy typically yield denser, less oxidized, higher-bond-strength coatings [39,40]. Cold spray was included as a solid-state alternative (already used at industrial scale for accident-tolerant fuels), in which particle bonding via adiabatic shear instability avoids melting-related oxidation while keeping substrate temperatures below the threshold at which recovery or recrystallization of cold-worked substrates could occur [41,42], isolating the effect of thermal input on coating protectiveness. Cr electroplating, finally, is a low-temperature, non-line-of-sight, conformal process governed by electrochemical reduction rather than particle impact [37,38], providing a bonding mechanism fundamentally different from the three spray routes. Diffusion-based routes (pack cementation) and vapor-phase techniques (CVD/ALD/PECVD, PVD/HiPIMS) were deliberately excluded from this paper, as their substantially different thermal budgets, growth mechanisms, and geometric constraints would not be directly comparable within the same experimental matrix. Such coating deposition methods are addressed in the second part of this study.

Overall, this coherent material–technology framework provides the technologically diverse basis required for the horizontal screening presented in this work, and a first-order rationale for down-selecting candidates toward component-specific qualification.

After presenting and discussing the experimental results, this paper provides initial considerations on the practical implementation of these coating technologies on representative lead fast reactor components. Attention is given to the implications of the observed corrosion behavior, technological maturity, scalability, and expected constraints associated with component geometry and service conditions.

Finally, results are discussed in the broader perspective of a future pre-qualification and qualification roadmap. These considerations are intended to identify the key experimental steps, performance indicators, and technology-specific challenges that should be addressed to qualify coatings as viable surface modification solutions for lead fast reactor components.

## 2. Materials and Methods

The substrates used in this study were primarily 316L(N) and 316Ti stainless steel plates cut by EDM to dimensions of 10 mm × 30 mm × 3 mm. This substrate variation is considered acceptable within the scope of the present screening campaign, as this study is focused on the chemical compatibility, integrity, and barrier effectiveness of the coating systems in liquid lead. In this respect, the substrate mainly acts as a mechanical support for the deposited layer, while the interaction with liquid lead is expected to be governed primarily by the exposed coating surface, its chemical composition, microstructure, density, continuity, and defect population. Therefore, provided that the coating remains continuous and sufficiently adherent, the underlying substrate is not expected to play a dominant role in the lead/coating interaction. Moreover, both 316L(N) and 316Ti are austenitic stainless steels with broadly comparable metallurgical and thermo-mechanical characteristics, thus providing similar support conditions for the investigated coatings. Although minor differences in coating/substrate interaction, interfacial bonding or residual stress cannot be fully excluded, the use of 316Ti or of 316L(N) is not expected to introduce major variations in deposition quality, provided that comparable surface preparation, roughness and cleaning procedures are applied prior to coating deposition.

All plates were labelled for traceability and subjected to surface preparation, ranging from sandblasting to mechanical polishing using SiC papers and diamond suspensions, in order to achieve the surface roughness required by each coating technology, from approximately Ra 7 µm down to Ra 0.1 µm. Prior to deposition, all substrates were cleaned in an ultrasonic bath with acetone to remove contaminants.

The coated samples investigated in this study were produced by selected external suppliers, allowing the screening campaign to include different deposition routes and coating systems, as detailed below.

**Atmospheric plasma spray (APS).** The APS process utilizes an electric arc discharge to ionize a gas mixture (typically Argon/Hydrogen or Argon/Helium), creating a thermal plasma jet. The feedstock powder is injected into this high-temperature jet, where particles are melted and accelerated toward the substrate. Key process parameters optimized by the supplier for this study included:

Stand-off distance: Maintained between 102 mm and 140 mm depending on the material.

Powder feed rate: Ranging from 42 g/min to 79 g/min.

Substrate Preparation: To ensure mechanical interlocking and adhesion, substrates were sandblasted to achieve a target surface roughness (Ra) of approximately 7 µm.

Temperature Control: Substrate temperatures were kept between 100 °C and 200 °C during deposition to mitigate residual stresses.

The materials deposited by APS within this screening campaign included the commercially available AFF FeCrAlY powder Amdry™ 9700 and a proprietary Alumina-Forming Austenitic (AFA) steel powder developed within *new*cleo’s materials R&D activities. Throughout the manuscript, this commercial FeCrAlY composition is referred to as AFF (alumina-forming ferritic alloy), with Y representing a minor reactive-element addition to the Fe–Cr–Al base composition.

The nominal chemical composition of the AFA powder, reported in Table 1—Nominal chemical composition of the proprietary AFA steel powder used for APS deposition, consists of Fe as balance, with Ni, Cr, Mn and Al as main alloying elements and minor additions of Nb, Ti, Si, C, S, O and N. This composition is covered by *new*cleo patent/application No. WO/2025/262632.

**Cold gas spray (CGS).** CGS was employed as a solid-state deposition technique, wherein feedstock powder particles are accelerated within a supersonic flow of inert gas, typically Nitrogen (N_2_). Impacting the substrate at velocities ranging from 300 to 1200 m/s, the particles undergo severe plastic deformation, triggering a physical phenomenon known as “adiabatic shear instability” [42]. This instability allows for metallurgical bonding and the subsequent coating build-up process while preserving the original microstructural and physical properties of the feedstock materials, effectively avoiding the thermal degradation and oxidation typical of high-temperature spray methods [43]. The deposition trials were performed utilizing an Impact 5/11c cold spray system produced by Impact Innovation GmbH. This high-pressure equipment allowed for precise optimization of particle velocity (Vp) and particle temperature (Tp) by preheating the propellant gas up to 700 °C and applying pressures up to 3.5 MPa. These conditions ensured that particles reached the critical velocity required for successful adhesion [44,45]. Two metallic alloy systems were investigated for CGS deposition: a commercial AFF FeCrAlY powder, Amdry™ 9700 supplied by Oerlikon, with a nominal particle size distribution of 45 ± 11 µm, and a custom-designed Alumina-Forming Austenitic (AFA) steel powder with a particle size distribution of 15–65 µm. Both powders exhibited a predominantly spherical morphology, selected to promote powder flowability and stable feeding during deposition. The AFA steel powder composition, reported in Table 2, is a proprietary *new*cleo formulation covered by *new*cleo patent No. WO/2025/262632. Owing to the intrinsically low thermal input of the CGS process, the substrate temperature was maintained below 200 °C during deposition, thereby avoiding the formation of a heat-affected zone (HAZ) and limiting the risk of thermally induced microstructural alterations in the base material.

**High Velocity-Oxy Fuel (HVOF).** The HVOF process accelerates feedstock particles through a high-pressure oxy-fuel jet to produce dense, highly deformed splats on the substrate. This HVOF deposition campaign targeted AFF FeCrAlY (AMDRY 9700) coatings and was carried out using a kerosene-fueled TAFA JP5000 torch in radial powder injection mode. An initial matrix of trials (varying O_2_:fuel ratio, carrier gas flow, stand-off distance and powder feed rate) was used to identify stable conditions that maximize particle fusion and coating density while avoiding nozzle clogging. Key parameters controlling particle temperature, velocity, and deposition quality were tuned for the two powders used (AFA and AFF).

Main parameter ranges used:

- Nozzle diameter: 101.6 mm.
- O_2_ flow: 800–900 slpm.
- Fuel (k1) flow: 0.28–0.35 L/min.
- Argon powder carrier: 8–12 slpm.
- Combustion pressure: 6.0–7.0 bar.
- O_2_ supply pressure: 7.5–8.5 bar.
- k1 supply pressure: 7.0–8.0 bar.
- Stand-off distance: 300–380 mm.
- Powder feed speed: 200–300 rpm

The corresponding powder compositions were the same as those described above for the CGS deposition route, namely the commercial AFF FeCrAlY Amdry™ 9700 and the proprietary *new*cleo AFA steel formulation reported in Table 2.

**Electroplating—Cr.** Electrodeposited chromium coatings with nominal thicknesses of 5, 25, and 120 μm. Electroplating relies on the electrochemical reduction of chromium-containing species from an electrolyte onto a conductive substrate, which is polarized as the cathode during the process. Under the applied current, chromium is progressively deposited on the activated substrate surface, while coating growth is governed by bath chemistry, current density, temperature, and deposition time. Prior to plating, the substrates were carefully cleaned, degreased and activated to remove surface contaminants and native oxides, ensuring suitable surface reactivity and promoting coating adhesion. Chromium electroplating was carried out in hexavalent chromium-based baths at temperatures below 60 °C, resulting in deposition rates of approximately 20–25 μm h^−1^. The different target thicknesses were obtained by adapting the plating duration.

### 2.1. Static Corrosion Tests

**Figure 1:**
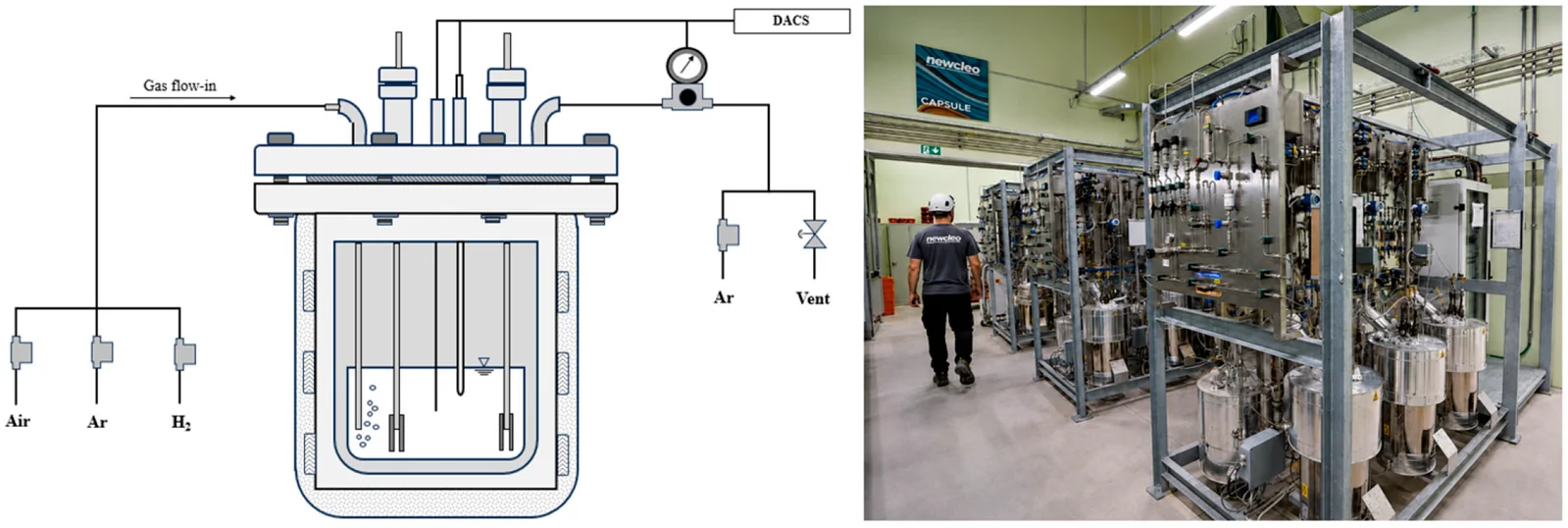
Scheme of CAPSULE setup (**left**) and picture of CAPSULE facility (**right**).

The exposure tests in liquid lead were carried out using *new*cleo’s CAPSULE facility at the ENEA Brasimone Research Centre, which is specifically designed for corrosion experiments under static, controlled liquid-metal conditions [20]. CAPSULE (Figure 1) is a facility for corrosion experiments in stagnant liquid lead. It consists of 6 skids, each containing 3 capsule-storage tank pairs. Every pair contains approximately 1 L of Pb, which can be transferred by overpressure between a conditioning vessel and the capsule where the corrosion test is performed. Each capsule consists of an alumina crucible housed in a stainless-steel enclosure and appropriately insulated. Up to six samples can be mounted simultaneously in each capsule.

Heating is provided by electrical heating elements mounted on the capsule, allowing a maximum operating temperature of 750 °C, while the oxygen content is adjusted by continuously bubbling a mixture of argon, hydrogen, and air (to increase and decrease the oxygen level) into the lead.

**Figure 2:**
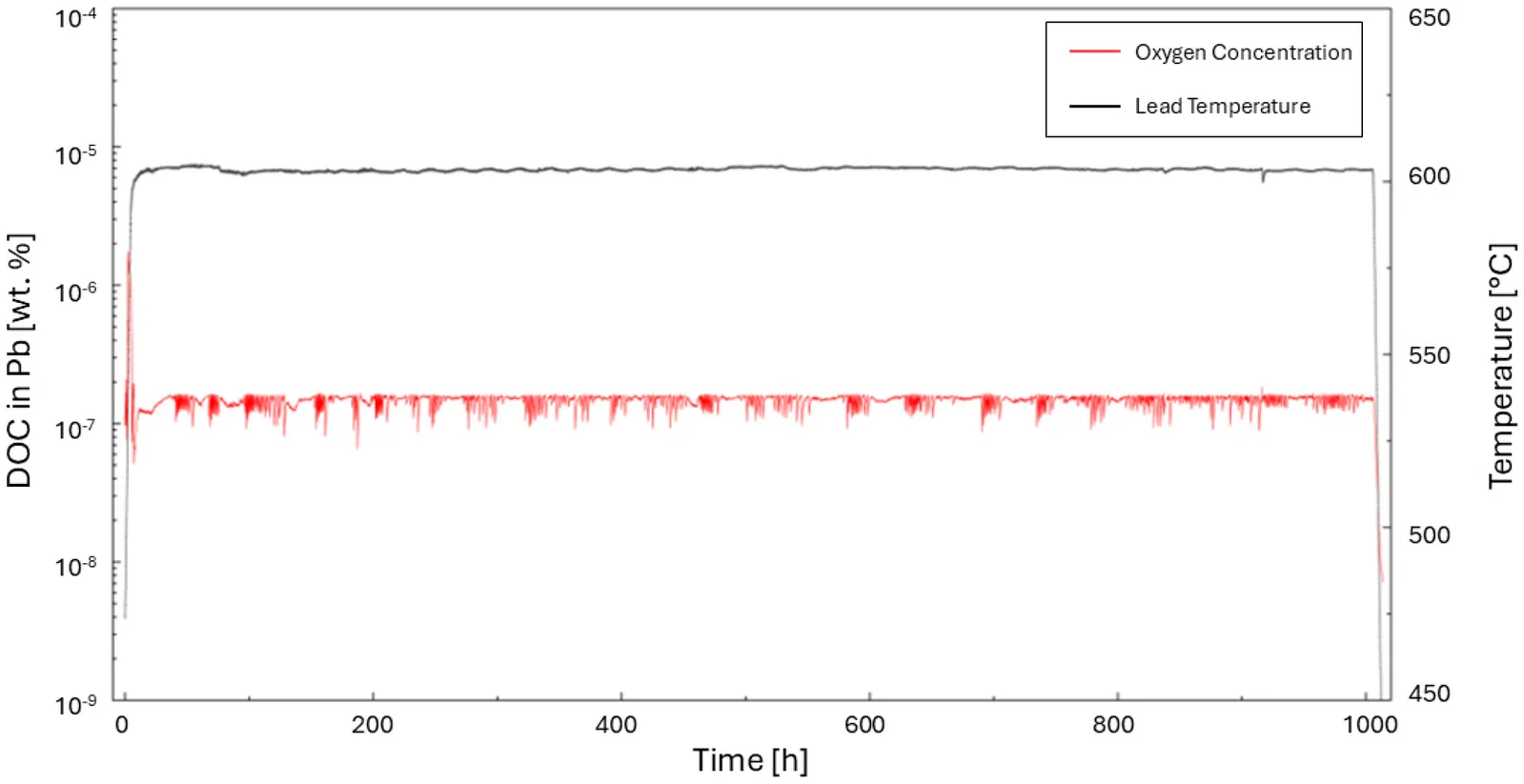
Representative time profiles of Pb temperature and dissolved oxygen concentration recorded during a liquid-lead exposure test in the CAPSULE facility. The profiles confirm that the target exposure conditions of 600 °C and 10^−7^ wt.% oxygen were maintained for 1000 h.

During each liquid-lead exposure test, both the Pb temperature and the dissolved oxygen concentration were continuously monitored in order to verify the stability and reproducibility of the test conditions. Figure 2 reports the temperature and oxygen concentration profiles recorded during a representative exposure test.

After the initial heating and stabilization stage, the system was maintained close to the target conditions of 600 °C and 10^−7^ wt.% oxygen for the full exposure duration of 1000 h. For reference, an oxygen concentration of wt.% corresponds to 1 ppb by mass.

The limited fluctuations observed during the test confirm the effectiveness of the control strategy adopted in the CAPSULE facility. All the exposure tests discussed in this work were carried out using the same experimental procedure and target conditions. The profiles shown in Figure 2 are representative examples of the overall testing campaign.

The liquid-lead exposure procedure was carried out according to a standardized sequence. First, the coated specimens were mounted on dedicated sample holders, which were then assembled inside the capsule and subjected to leak testing. The internal volume of the capsule was subsequently purged with argon to establish an inert atmosphere. In parallel, the lead was conditioned in the storage vessel, while the capsule was heated to the transfer temperature of approximately 400 °C. Once the lead was molten, it was transferred from the storage vessel to the capsule. The system was then brought to the target exposure conditions in terms of temperature and dissolved oxygen concentration. After stabilization, the samples were immersed in the liquid-lead bath, marking the start of the exposure time, while temperature and oxygen concentration were continuously monitored throughout the test.

For each coating material–technology combination, two nominally identical specimens were exposed under the same test conditions. Following exposure, both specimens were visually inspected to verify consistency of the overall response to liquid Pb, particularly in terms of coating integrity, macroscopic degradation, and evidence of substrate protection or attack. Detailed cross-sectional characterization and the maximum corrosion or Pb-penetration depths reported in this work were subsequently obtained from one representative specimen per condition. This approach is consistent with the exploratory nature of the present horizontal screening, whose primary objective is the initial down-selection of candidate coating systems. More extensive replication and statistical characterization will be performed on the most promising systems during subsequent qualification-oriented testing.

At the end of the experiment, the capsule was cooled to the transfer temperature of approximately 400 °C, while maintaining the controlled inert atmosphere, and the Pb was then drained to the storage tank. The inert atmosphere was maintained throughout the subsequent cooling stage; air was admitted only during specimen loading and unloading, when the system temperature was below approximately 100 °C.

The rods with the samples are extracted once the capsule has cooled down to room temperature. Lead residuals are washed from the surface of the samples, with a few exceptions to demonstrate the presence of Pb in cross-section analysis. Washing of the samples is carried out with a solution for 24 h, composed of the following parts: (i) 1/6 acetic acid (CH_3_(COOH)), which forms Pb acetate; (ii) 1/6 ethanol (C_2_H_6_O), which slows down the reaction; (iii) 1/6 hydrogen peroxide (H_2_O_2_), which enhances the oxidizing effect of the solution; (iv) 1/2 demineralized water (H_2_O), to dilute the solution. This procedure has been routinely applied in liquid-Pb testing and has shown no evidence of cleaning-induced modification of the coating morphology, coating/substrate interface, or stable oxide-rich regions during subsequent metallographic and SEM-EDS characterization.

The samples are finally prepared for cross-section analyses by cutting, embedding in conductive resin, and polishing with diamond paste to obtain a mirror-finished morphology.

### 2.2. Surface Mechanical and Microstructural Characterization

Samples are analyzed in *new*cleo’s advanced characterization facility, established within Turin’s Environment Park as part of a public-private partnership with Istituto Italiano di Tecnologia (CoSyET SCARL). SEM-EDS characterization was performed using a ZEISS GeminiSEM 360 field-emission scanning electron microscope equipped with an Oxford Instruments Ultim Max 100 energy-dispersive X-ray spectroscopy detector. The same analytical setup and workflow were adopted before and after liquid-lead exposure to ensure a consistent comparison between the as-deposited and exposed conditions. Prior to exposure, the coated samples were analyzed in plan view at different magnifications to assess the initial surface morphology and elemental distribution. Selected specimens were also sectioned, mounted, and polished for cross-sectional SEM-EDS analysis, in order to evaluate coating thickness, internal microstructure, defect population, and coating/substrate interface quality.

After liquid-lead exposure and cleaning, all samples were characterized following a standardized post-corrosion SEM-EDS workflow, aimed at identifying degradation features and enabling a consistent comparison among the different coating systems. Each specimen was first inspected at low magnification by optical microscopy over the entire exposed surface. This macro-scale survey was used to detect evident signs of post-exposure degradation, including coating detachment, delamination, cracking, localized corrosion attack, residual lead deposits, and regions showing anomalous surface morphology.

Following this preliminary inspection, plan-view SEM analyses were performed on four representative areas of each sample using predefined magnifications. These observations were used to assess the exposed surface morphology and to identify local features such as surface oxides, coating discontinuities, lead residues, corrosion products, or compositional heterogeneities. SEM imaging was systematically coupled with EDS point analyses and elemental mapping to evaluate the distribution of the main coating elements, oxygen and lead, and to support the interpretation of possible coating–lead interactions.

For cross-sectional characterization, the specimens were sectioned along their longitudinal direction, i.e., parallel to the long side of the sample. This cutting strategy was adopted to maximize the length of the exposed coating/substrate interface available for observation, thereby increasing the inspected area and improving the probability of detecting localized degradation phenomena such as lead penetration, coating discontinuities, delamination, interface attack, or localized corrosion fronts. The selected sections were then mounted and polished to obtain a suitable surface finish for SEM-EDS analysis.

For each cross section, four regions of interest were analyzed by SEM-EDS. The analyzed regions were selected to include both apparently intact areas and locations where the plan-view inspection suggested possible degradation. Cross-sectional observations were used to evaluate coating integrity after exposure, residual coating thickness, internal cracking or porosity, coating/substrate interface stability, elemental redistribution, oxide formation, corrosion depth, and possible penetration through the coating or into the underlying substrate.

This combined macro-scale, plan-view and longitudinal cross-sectional approach allowed the post-corrosion behavior of each coating system to be assessed in a reproducible and comparable manner, while maximizing the inspected surface and reducing the risk of overlooking localized degradation features.

Coating thickness and degradation depths were measured on polished cross sections using SEM images acquired at standardized magnifications. Coating thickness was defined as the perpendicular distance between the outer coating surface and the coating/substrate interface. For each specimen, thickness measurements were performed in representative regions, excluding edges and evident preparation artefacts, and reported as average values with the corresponding dispersion where applicable.

To distinguish actual substrate corrosion from degradation confined within the coating, two depth-related parameters were defined. Maximum corrosion depth was used only when the degradation front reached the substrate. In this case, it was defined as the largest perpendicular distance from the original substrate surface, or from the original coating/substrate interface in coated regions, to the deepest corrosion front or lead-affected zone observed within the substrate.

When no substrate corrosion was observed, degradation was instead described in terms of maximum Pb penetration depth within the coating. This parameter was defined as the largest perpendicular distance from the exposed coating surface to the deepest location at which Pb was directly detected within the coating by SEM-EDS. This parameter is descriptive and does not, by itself, identify the underlying transport mechanism. Where Pb ingress was spatially associated with pre-existing cracks, pores or other coating discontinuities, it was interpreted as defect-assisted infiltration; otherwise, no distinction between bulk diffusion and transport along unresolved microstructural pathways was made. Chemical interaction or elemental redistribution without direct evidence of Pb ingress was considered separately.

This distinction ensures that coating-limited lead penetration is not misinterpreted as substrate corrosion when the bulk material remains protected.

Both maximum corrosion depth and maximum lead penetration depth were used as conservative indicators of the most severe local degradation observed within the analyzed cross section, rather than as average corrosion rates. Edge regions were systematically excluded from quantitative measurements, since sample corners and edges may promote non-representative coating build-up, local stress concentration, preferential lead access, polishing artefacts or enhanced degradation. Measurements were therefore performed only in central regions of the exposed surface, where the coating morphology and corrosion behavior were considered representative of the investigated material–technology combination.

Ancillary mechanical and microstructural characterization was performed on representative as-deposited coatings to support the assessment of deposition quality and to provide complementary information for the interpretation of the liquid-lead exposure results. The analyses included cross-sectional hardness measurements, porosity evaluation, and metallographic inspection of the coating/substrate interface, with particular attention to coating consolidation, defect population, and possible deposition-induced substrate modifications.

Vickers microhardness measurements were carried out on polished cross sections according to ASTM E92-17/ISO 6507-1. The applied load was selected according to coating thickness and microstructural scale, in order to obtain representative indentations within the coating while minimizing the influence of neighboring features or of the substrate. For spray coatings, hardness was generally measured both within the coating and in the adjacent substrate, avoiding edges, pores, cracks, delaminated regions, and polishing artefacts. When multiple valid indentations were available, results were reported as average values with the corresponding standard deviation.

For chromium electrodeposited coatings, the mechanical testing procedure was adapted to the coating thickness. Conventional Vickers microhardness was used for thicker deposits, whereas nanoindentation with a Berkovich indenter, according to ASTM E2546, was used for thinner coatings, where standard microhardness measurements would be strongly affected by substrate contribution. Where required, nanoindentation results were converted into Vickers-equivalent values to allow comparison with the other coating systems.

Porosity was estimated from polished cross-sectional SEM micrographs using grayscale threshold-based segmentation of representative coating areas. The segmented pore area was normalized to the total analyzed coating area and is therefore reported as an apparent two-dimensional pore-area fraction, rather than as a direct measurement of three-dimensional total or open porosity. The number of analyzed fields, field size, and image magnification were not standardized across all coating systems, as the porosity data were obtained within coating-specific characterization campaigns. Such measurements are inherently sensitive to local microstructural heterogeneity, section position, pore morphology, image scale and segmentation criteria, particularly when comparing coatings produced by different deposition mechanisms and characterized by different pore shapes, orientations and spatial distributions. Accordingly, the reported values are used as semi-quantitative descriptors of coating compactness and defect population, interpreted together with the corresponding cross-sectional observations, and not as statistically equivalent stand-alone ranking parameters across deposition technologies.

Etched cross sections were also inspected by optical microscopy and SEM to assess the thermally affected zones, localized recrystallization, grain growth, interfacial discontinuities, or other deposition-induced microstructural changes in the substrate. This was particularly relevant for comparing high-thermal-input processes, such as APS, with lower-thermal-input or solid-state routes, such as HVOF and CGS.

Part of the mechanical and microstructural characterization was performed directly by the coating suppliers as part of their process development and optimization activities. These data were used to support the interpretation of coating quality and process robustness, while the comparative post-exposure SEM-EDS characterization was performed within the common framework adopted in this study. Because the primary objective of the work was liquid-lead compatibility, the mechanical dataset was considered ancillary and semi-quantitative. Accordingly, differences in the completeness of the available mechanical data among coating variants were explicitly considered, and these data were not used as an independent ranking criterion.

The coating–substrate interface assessment by optical microscopy and SEM was qualitative and was intended to identify interfacial discontinuities, delamination, unbonded regions and deposition-induced substrate modifications. These observations were not used as a quantitative measure of coating adhesion or bond strength.

Post-exposure mechanical testing, adhesion testing, wear testing, fatigue assessment and thermal-cycling experiments were not included in this first screening campaign. These tests were deliberately deferred to subsequent qualification-oriented studies because they require larger sample statistics, dedicated specimen geometries and loading parameters specific to each component and its operating conditions (both normal and transient or accidental). The present mechanical characterization was therefore limited to the as-deposited condition and used only to support the interpretation of coating density, hardness, interface quality and possible substrate alteration prior to corrosion exposure.

## 3. Results

This section shows the results of the investigation structured by the coating technology-material couples investigated. Namely, APS of AFA and AFF steel, CGS of AFA and AFF steel, electrochemical deposition of Cr, HVOF of AFA and AFF steel. The results are gathered systematically into image compositions comprising top- and cross-sectional views before and after corrosion tests with the relative EDX maps, as well as macro-stitches of sample cross-sections.

### 3.1. APS AFA

Quantitative image analysis on five fields returns porosity values of 1.98–3.13 vol.% (average 2.64 vol.%), confirming a low but measurable intersplat/microporosity. Vickers microhardness (ASTM E92-17) on the optimum coating (0.1 kg load) yields an average of 254 HV0.1 (std. dev. 48.4); substrate hardness measured with 0.5 kg load is 154.8 HV0.5 (std. dev. 15.8). Chemical etching and optical inspection reveal substrate microstructure changes in the interfacial zone: a heat-affected zone (TAZ) extending 25 µm from the interface, characterized by localized recrystallization. These features oxide-rich interparticle regions, limited microporosity, elevated coating hardness, and a shallow TAZ will affect mechanical behavior and interfacial reactions and should be considered when comparing APS-AFA performance with other deposition routes (e.g., HVOF, cold spray).

**Figure 3:**
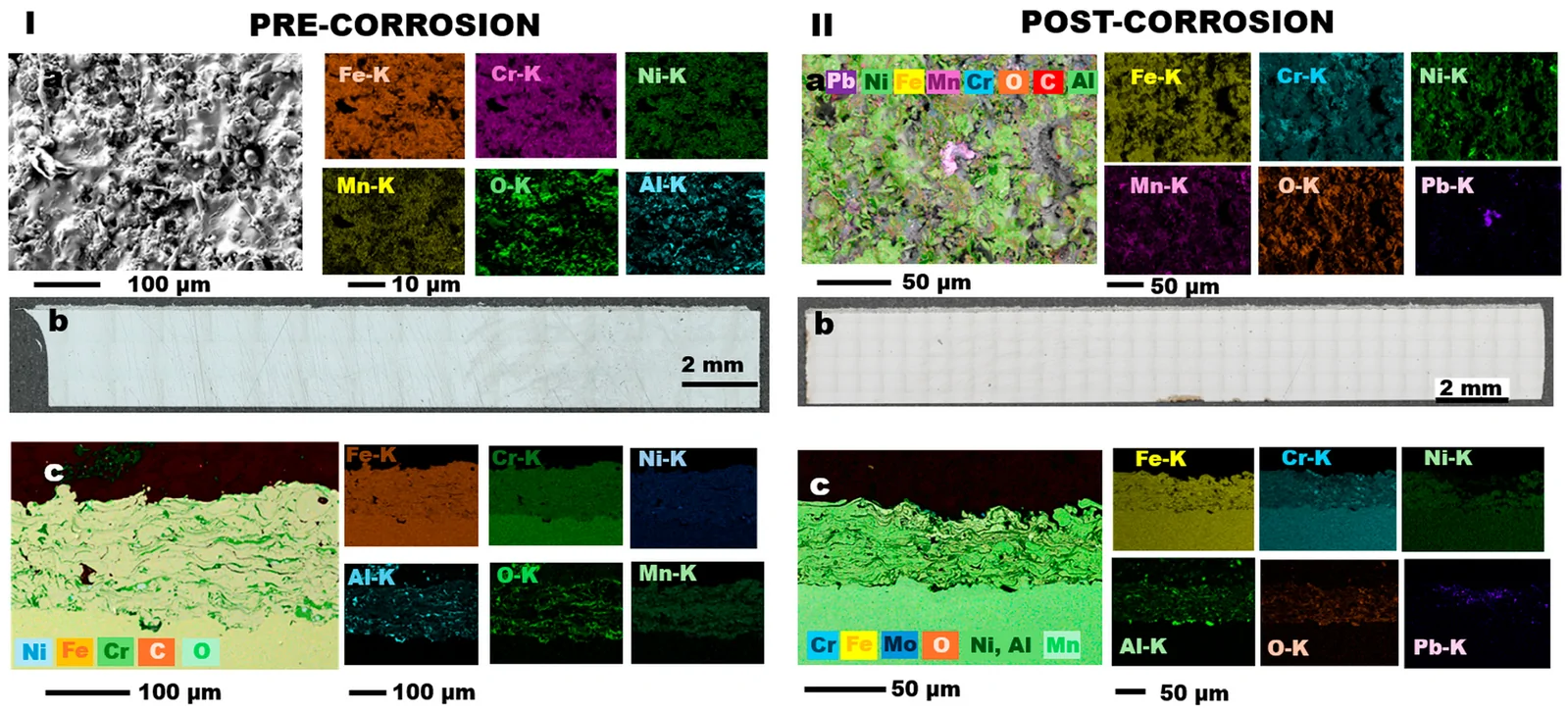
Microscopy analyses of APS AFA samples pre-corrosion (**I**) and post-corrosion (**II**), including SEM-EDS maps in top view (**a**) and in cross-sectional view (**c**), as well as cross-sectional optical macros (**b**).

Surface analyses pre-corrosion (Figure 3(Ia)) show the deposition surface, for which the morphology is commonly found in APS coatings. The cross-sectional analyses pre-corrosion (Figure 3(Ic)) reveal the typical microstructure of APS coatings too, which includes splats, pores, lamellar structures, and crack networks. The thickness of the coatings is in the range of 142.1 ± 3.5 µm. The SEM-EDS maps indicate a similar spatial distribution of aluminum and oxygen, suggesting some oxidation during deposition, which is typical in APS processes given the high temperatures involved (e.g., particles impact the substrate at 2000–3000 °C).

The corrosion test in liquid lead did not result in corrosion of the underlying steel. However, the SEM EDS (Figure 3(IIa,IIc)) and optical microscope (Figure 3(IIb)) analyses reveal an interaction of the liquid metal with the coating in specific locations, where an enrichment with Mn is apparent. The cross-sectional SEM-EDS observations (Figure 3(IIb,IIc)) show that lead interacted with the coating material in such locations. In most regions, however, the coating remained intact, and the substrate was effectively protected. SEM-EDS detected Pb locally within the coating, with a maximum penetration depth of approximately 62.9 µm. No corrosion was observed on the underlying coated substrate. The uncoated surface of the steel sample (bottom in Figure 3(IIb)) displays corrosion attacks.

### 3.2. APS AFF

The substrate region directly beneath the APS coating showed no evidence of melting. However, a shallow thermally affected zone (TAZ), extending approximately 25 µm from the coating/substrate interface, was observed. This region was characterized by localized recrystallization and microstructural variations, indicating that the thermal input during APS deposition was sufficient to modify the near-interface substrate microstructure without reaching the melting regime. Cross-sectional image analysis performed on five representative fields yielded porosity values between 0.82 and 4.06 vol.%, indicating limited but measurable porosity within the APS coating. The coating thickness was 99.50 ± 3.93 µm. Vickers microhardness (ASTM E92, 0.1 kg) of the optimum APS coating is 236 HV0.1 (std. dev. 20.7); substrate hardness (HV0.5) is 154.4 (std. dev. 16), with increased hardness in the interfacing area consistent with the observed microstructural changes.

**Figure 4:**
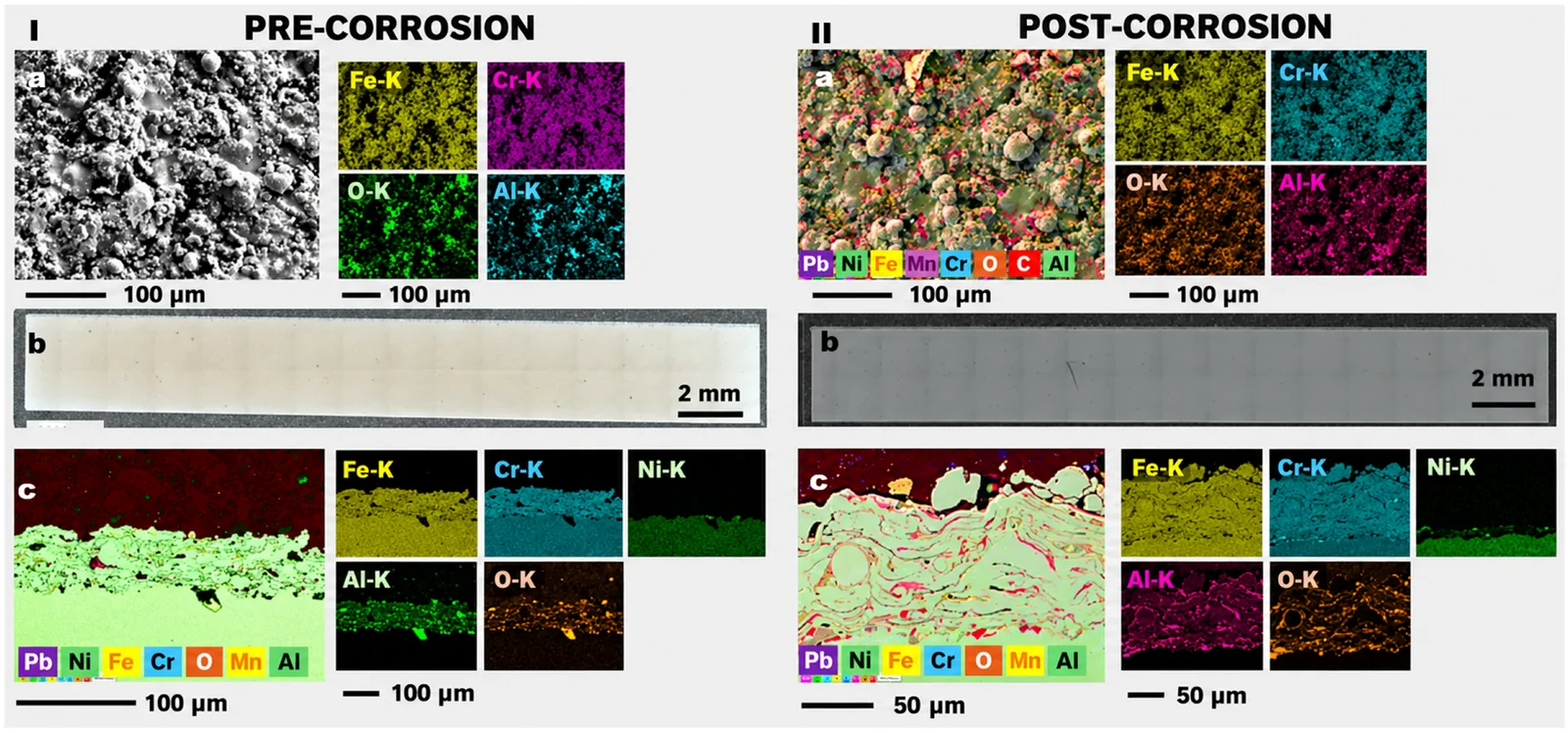
Microscopy analyses of APS AFF samples pre-corrosion (**I**) and post-corrosion (**II**), including SEM-EDS maps in top view (**a**) and in cross-sectional view (**c**), as well as cross-sectional optical macros (**b**).

SEM and EDS analysis of APS-deposited AFF coatings show, in top-view images before corrosion testing (Figure 4(Ia)), a surface covered by aluminum oxide patches attributable to air atmosphere plasma spraying; the particle morphology is less dominated by intact spherical imprints than in HVOF, reflecting melting and solidification effects typical of APS. Cross-sectional SEM (Figure 4(Ic)) reveals more pronounced splat spreading and interlayer consolidation with clear evidence of oxidation at particle–particle interfaces and detectable microporosity within the deposit.

After exposure to molten lead and subsequent cleaning, top-view imaging (Figure 4(IIa)) confirms the presence of discrete oxide spots and continuous oxide regions that act as protective scales against corrosion. Cross-sectional inspection post-test shows no observable Pb penetration or continuous Pb-ingress pathways through the coating, while EDS mapping confirms the pre-existing interparticle aluminum oxide phases within the layered structure—features that were already noted in the as-sprayed APS condition and that, for comparison, will be discussed alongside the HVOF results presented subsequently in the manuscript.

### 3.3. CGS AFA

Cold-spray AFA coatings were developed through an iterative process-optimization campaign focused on coating adhesion, density and interface quality. The deposition trials showed that excessive thermal input promoted oxidation, residual-stress build-up and coating detachment, whereas insufficient particle deformation resulted in higher porosity and poor interparticle bonding. A second campaign, performed with a reduced number of deposition passes to limit coating thickness and deformation, identified an optimized processing window characterized by adequate particle acceleration and intermediate thermal input. This condition provided the best compromise between coating build-up, low porosity, and good coating/substrate interface quality. Porosity image analysis on two selected cross-section fields (gray-scale thresholding) gave an apparent porosity below 2.4 vol.%. Microhardness testing (ASTM E92-17) using a 0.5 kg Vickers load on the cross-section returned an average coating hardness of 377 HV0.5 (std. dev. 15.0); substrate bulk hardness averaged 149 HV0.5 (std. dev. 2.1). Chemical etching and microstructural inspection show no detectable substrate alteration at the interface (no TAZ, no recrystallization or grain growth). The optimized cold-spray AFA coatings were therefore characterized by limited but measurable porosity, high hardness and no detectable thermally induced substrate modification, indicating good coating consolidation compared with higher-temperature deposition routes.

**Figure 5:**
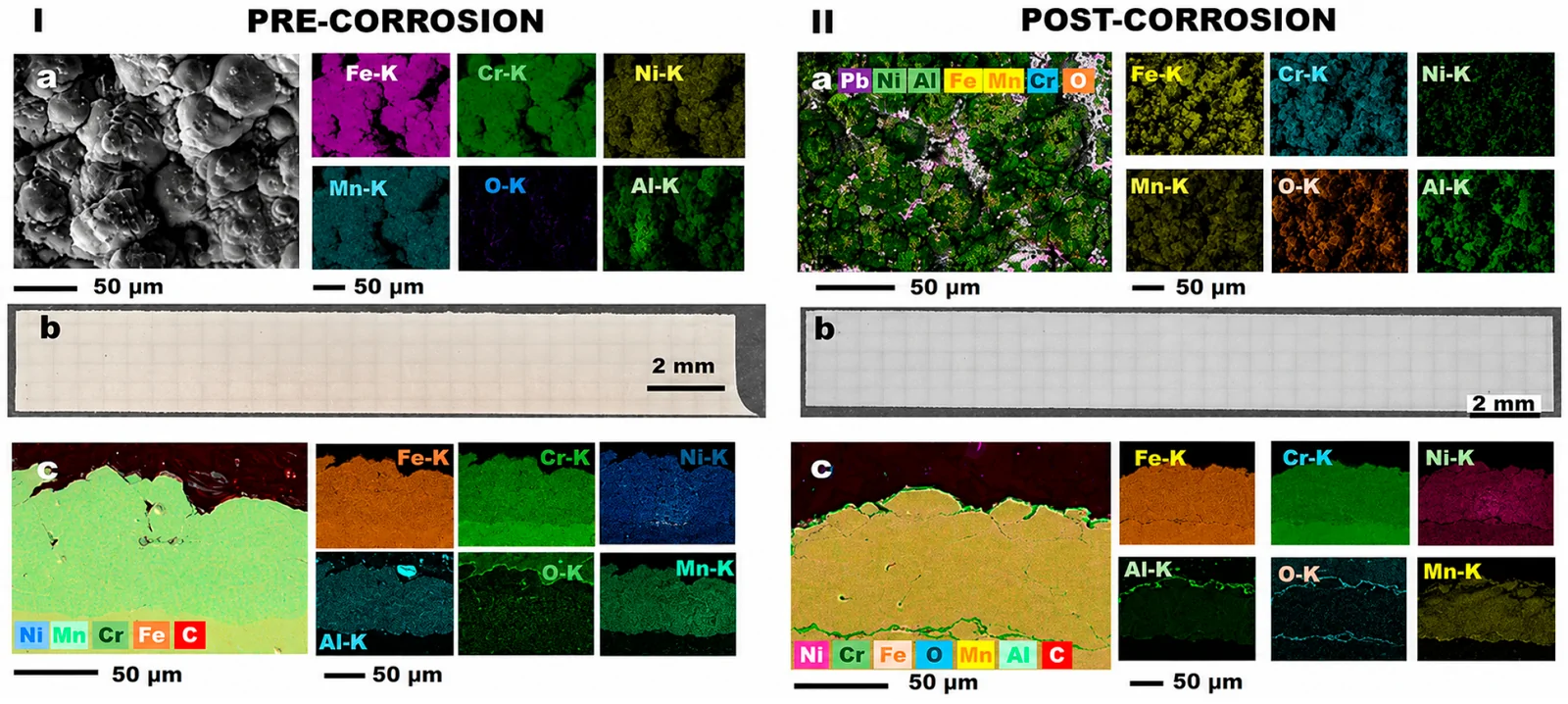
Microscopy analyses of cold-spray AFA samples pre-corrosion (**I**) and post- corrosion (**II**), including SEM-EDS maps in top view (**a**) and in cross-sectional view (**c**), as well as cross-sectional optical macros (**b**).

The surface analyses pre-corrosion, shown in Figure 5(Ia), show the deposition surface, from which the shape of the impinging particles is visible. The cross-sectional analyses (Figure 5(Ib,Ic)) reveal the typical microstructure of cold spray coatings. The microstructure is dense, despite occasional porosity resulting from mechanical deformation during the impact of the powder particles. The thickness of the coatings is in the range of 60 ± 4 µm. The SEM-EDS maps indicate homogeneous spatial distribution of the elements composing the AFA alloy.

The corrosion test in liquid lead did not result in corrosion of the underlying steel, as confirmed by the SEM (Figure 5(IIa,IIc)) and optical microscope analyses. The cross-sectional SEM-EDS observations (Figure 5(IIb,IIc)) show enrichment of Al and oxygen at the top surface of the coating, pointing towards the formation of protective Al-rich oxide scales. An enrichment of oxygen, chromium, and manganese is also found at the interface. A deeper investigation into the origins and consequences of the enrichment is excluded from the scope of this work.

### 3.4. CGS AFF

Optical/SEM and EDS analyses of the cold-sprayed samples show clearly the imprints and shapes of impacting powder particles on the deposition surface, and cross-sections reveal the characteristic dense, heavily deformed particle microstructure of cold spray with only occasional interparticle/interface voids. Image analysis gives apparent porosity < 0.9 vol.%. Vickers microhardness (ASTM E92-17, 0.5 kg) of the optimum coating averages 316 HV0.5 (std. dev. 21.8), while the substrate averages 157 HV0.5 (std. dev. 3.8). The process-optimization campaign showed that coating quality was strongly governed by particle impact conditions. Higher impact energy promoted particle deformation, improved interparticle bonding and reduced porosity, whereas low-energy conditions resulted in poor deposition efficiency and were excluded from further assessment. The optimized condition provided the best compromise between coating density, cohesion and deposition efficiency. Chemical etching and microstructural inspection confirm no detectable substrate microstructural change at the interface (no TAZ, no recrystallization or grain growth), consistent with the solid-state deposition mechanism.

**Figure 6:**
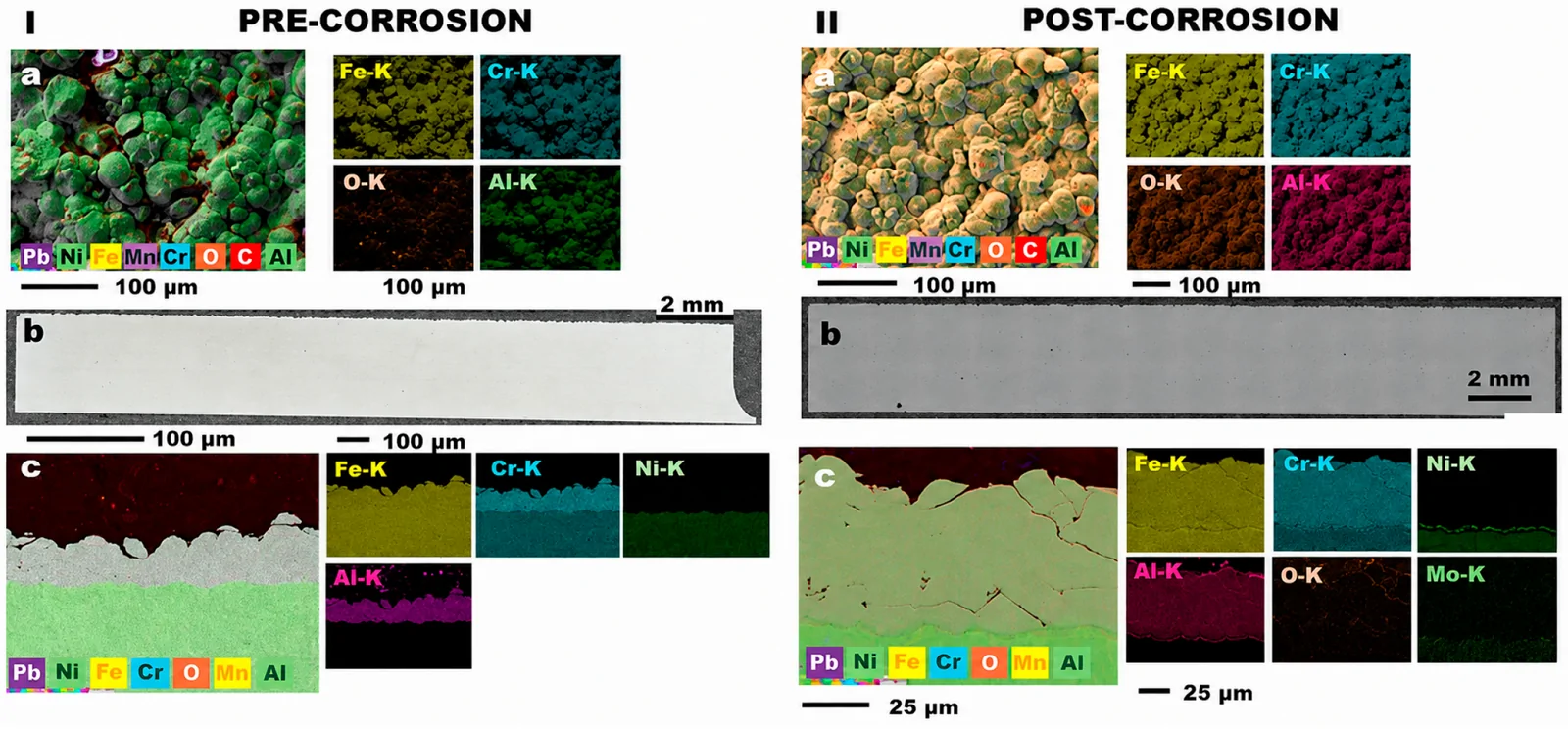
Microscopy analyses of cold spray AFF samples pre-corrosion (**I**) and post (**II**), including SEM-EDS maps in top view (**a**) and in cross-sectional view (**c**), as well as cross-sectional optical macros (**b**).

The deposition surface in the pristine condition is visible in Figure 6(Ia). The shape of the impacting powder particles is clearly visible. The cross-sectional analyses (Figure 6(Ib,Ic)) reveal the typical microstructure of cold spray coatings. The microstructure is dense. Pores and interface voids between impacting particles are found occasionally. The thickness of the coatings is in the range of 77 ± 5 µm. The SEM-EDS maps indicate homogeneous spatial distribution of the elements composing the AFF alloy.

The corrosion test in liquid lead does not result in corrosion of the underlying steel, as confirmed by the SEM (Figure 6 (IIa,IIc)) and optical microscope (Figure 6 (IIb)) analyses. The cross-sectional SEM-EDS observations (Figure 6(IIb,IIc)) show enrichment of Al and oxygen at the top surface of the coating, suggesting that a protective Al-rich oxide layer formed on the surface. Cross-sectional SEM-EDS analysis at the interface indicates nickel migration from the substrate toward the Al present in the AFA coating, creating a Ni depletion in the bulk material. Further investigation is needed to shed light on the origins of this migration and its consequences. Such investigation is out of scope of the present systematic horizontal screening.

### 3.5. Electrochemical Deposition Cr

**Figure 7:**
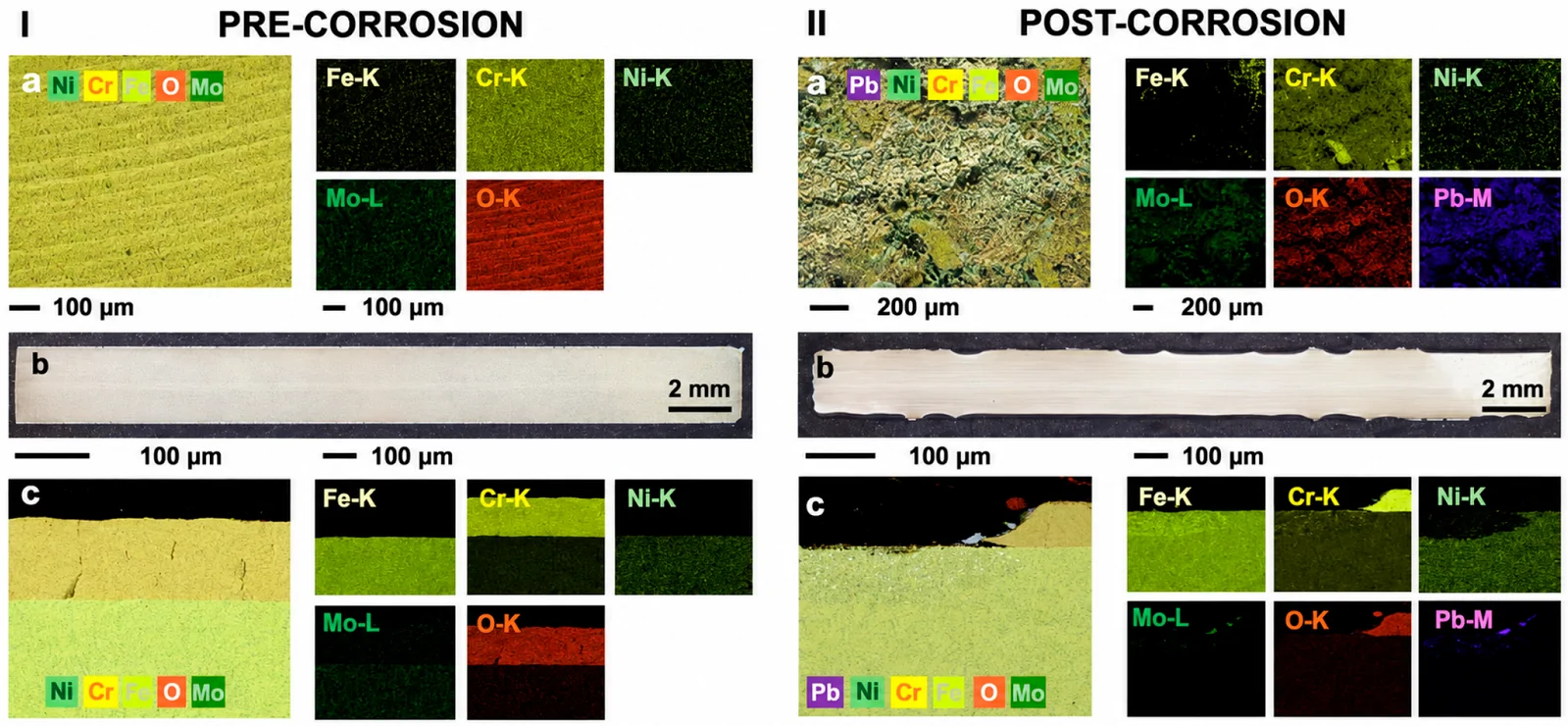
Microscopy analyses of Cr-electroplated samples pre-corrosion (**I**) and post-corrosion (**II**), including SEM-EDS maps in top view (**a**) and in cross-sectional view (**c**), as well as cross-sectional optical macros (**b**).

Surface analyses pre-corrosion (Figure 7(Ia)) show uniform depositions, while cross-sectional analyses (Figure 7(Ib,Ic)) revealed a dense network of microcracks, with their extent and connectivity increasing with coating thickness. Localized over-thickening of the Cr deposit was observed at sharp edges, as commonly expected in electrodeposition due to current-density concentration at geometrical discontinuities. EDS maps confirmed the chemical purity of the chromium coatings, with no detectable impurities or substrate contamination.

Cross-sectional hardness was evaluated by Vickers micro-hardness (ISO 6507-1, 0.1 kgf) and, for the thinnest coatings, by nanoindentation (ASTM E2546, Berkovich indenter, 2 mN) with results converted to Vickers equivalent where required. Measured hardness and mean thickness are summarized below (HV values are ranges/means as reported):

4.6 µm mean thickness: mean 720 HV (nanoindentation → Vickers equivalent).

24.2 µm mean thickness: mean 868 HV (nanoindentation → Vickers equivalent).

36.5 µm mean thickness: 613 HV (single value, HV0.1).

118 µm mean thickness: mean 920 HV (HV0.1).

Hardness increases with thickness in these samples, but the presence and connectivity of microcracks at larger thicknesses should be considered when assessing mechanical integrity and corrosion performance. Nanoindentation was required for the thinner deposits to limit substrate effects, while Vickers microhardness was used only where coating thickness allowed reliable indentation within the coating.

After exposure to liquid Pb, top-view SEM (Figure 7 (IIa)) showed extensive loss of the Cr coating, with only sporadic regions remaining on the substrate. The present post-exposure characterization does not allow the relative contributions of coating dissolution and detachment to this coating loss to be distinguished unambiguously. Cross-sectional observations show that the pre-existing microcrack network provided preferential pathways for Pb infiltration, allowing Pb to reach the underlying substrate. Actual substrate corrosion was subsequently observed, together with significant Ni depletion, with a maximum corrosion depth of approximately 280 µm.

### 3.6. High Velocity Oxy-Flame AFA

**Figure 8:**
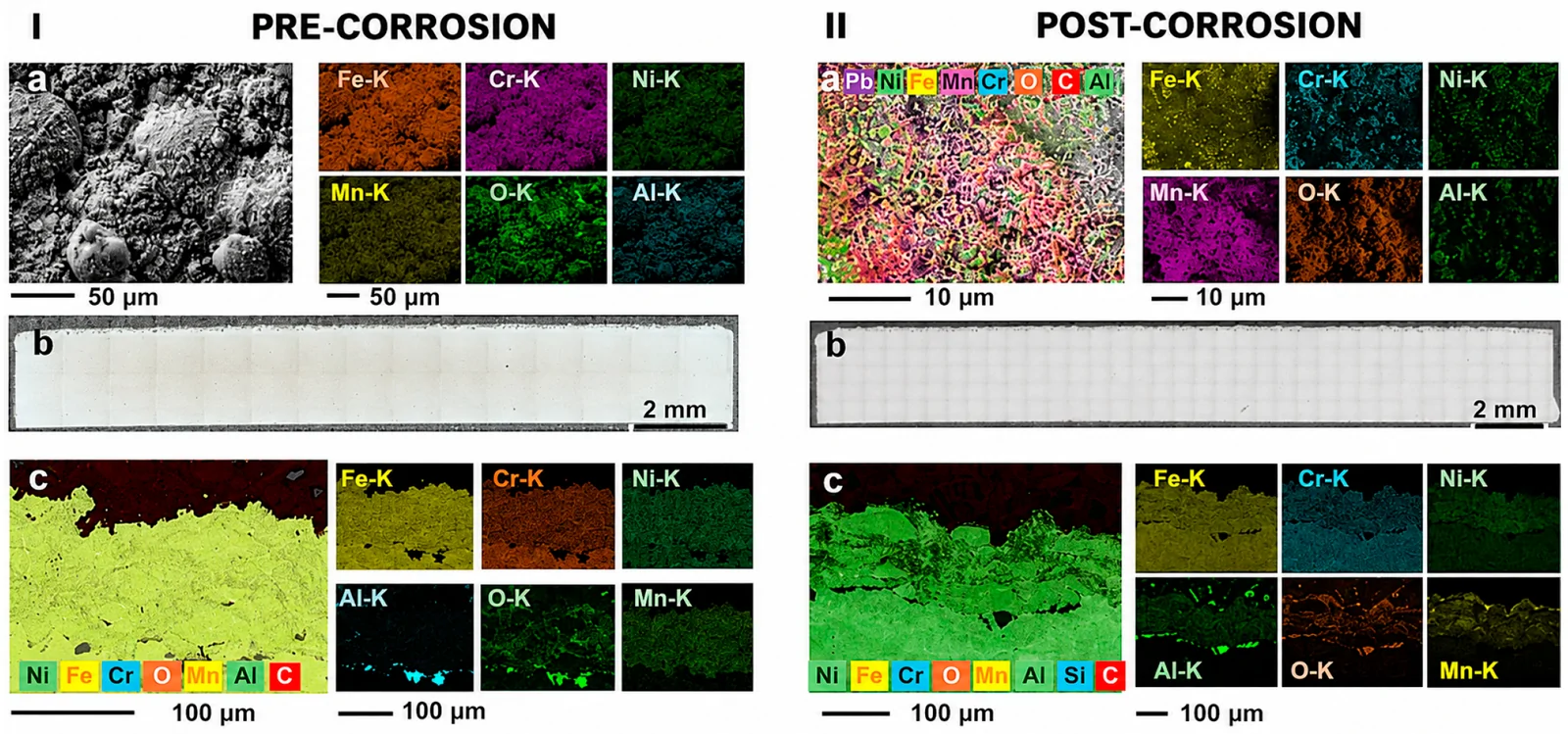
Microscopy analyses of HVOF AFA samples pre-corrosion (**I**) and post-corrosion (**II**), including SEM-EDX maps in top view (**a**) and in cross-sectional view (**c**), as well as cross-sectional optical macros (**b**).

A dedicated HVOF campaign was conducted to identify optimal processing for AFA powders using varied O_2_/fuel ratios, carrier gas flows, powder feed rates, and stand-off distances. SEM analysis (Figure 8(Ia)) of pristine HVOF-AFA surfaces shows the expected rough topography; cross-sections display a layered HVOF morphology with partially flattened splats, limited inter-splat porosity, and occasional microcracks (to a lesser extent than APS AFA). SEM-EDS maps indicate co-localization of Al and O, consistent with some in-flight oxidation during deposition. Optical and metallographic inspection give a coating thickness of 93.4 ± 3.9 µm and very low apparent porosity (image analysis average 0.65 vol.%). Vickers hardness measured on polished cross-sections (HV0.5, ASTM E92) is uniform through the layer, with a coating of average 326 HV0.5 (std. dev. 19.3) and a substrate bulk of 167.6 HV0.5 (std. dev. 33.8), indicating good particle consolidation and mechanical homogeneity. Chemical etching reveals no detectable substrate microstructural alteration near the interface (no grain growth or recrystallization) despite grit-blasting and preheating, and no delamination or unbonded regions were observed, confirming effective adhesion and process reliability under the selected HVOF conditions.

The steel substrate did not suffer any corrosion attack during the test, as confirmed by the surface SEM (Figure 8(IIa)) and optical microscope (Figure 8(IIb)) analyses. The cross-sectional SEM-EDS observations (Figure 8(IIc)) suggest that protective scales of complex chemical composition, including aluminum and manganese, formed on the surface of the coatings.

### 3.7. High Velocity Oxy-Flame AFF (FeCrAlY)

**Figure 9:**
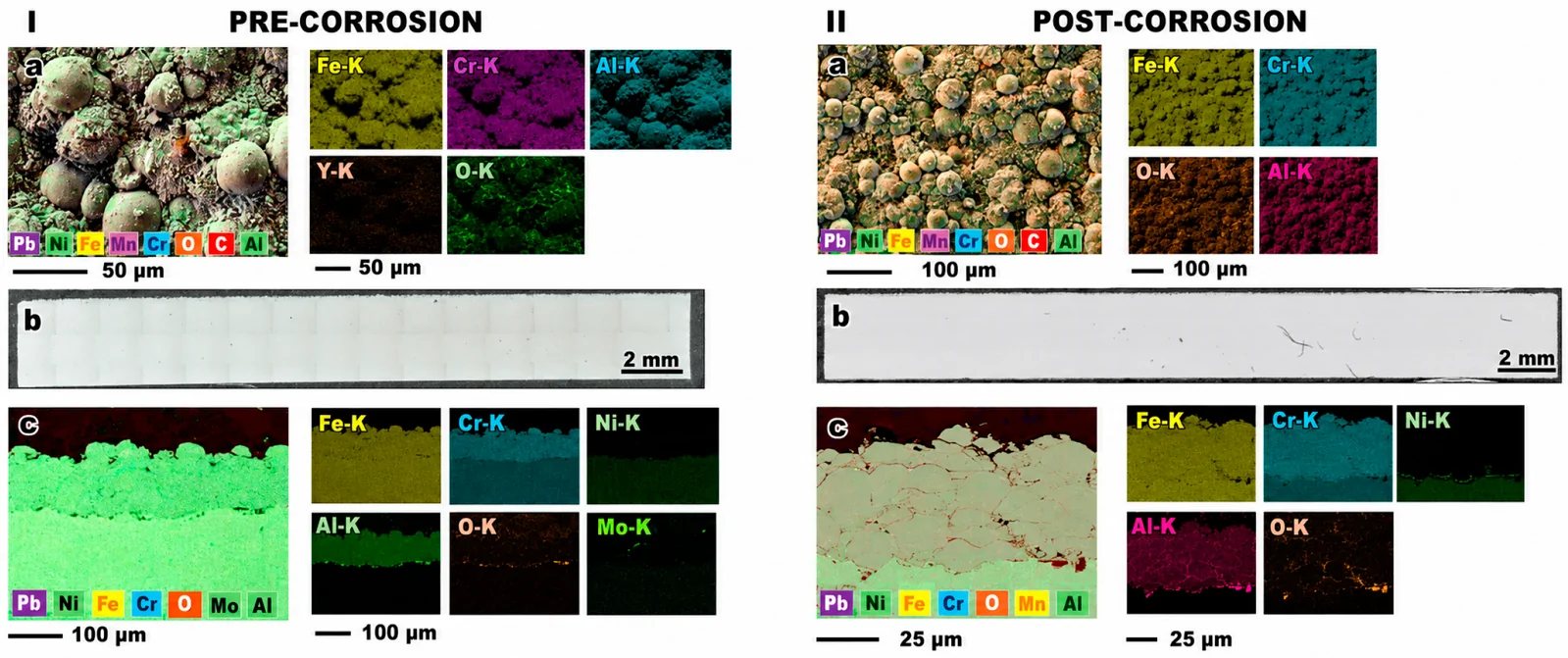
Microscopy analysis of HVOF AFF (FeCrAlY) samples pre-corrosion (**I**) and post-corrosion (**II**), including SEM-EDS maps in top view (**a**) and cross-sectional view (**c**), as well as cross-sectional optical macros (**b**).

SEM analysis of the HVOF-deposited AFF (FeCrAlY) coating reveals (Figure 9), in top-view images prior to corrosion testing, the characteristic spherical morphologies and particle outlines inherited from gas-atomized feedstock, together with partial flattening consistent with high-velocity impact. Micro-EDX mapping of the as-sprayed surface shows a thin, continuous-to-semi-continuous Al-rich oxide scale formed during air-atmosphere HVOF (AHVOF) deposition, while cross-sectional SEM displays the typical HVOF microstructure of plastically deformed splats and layered deposition bands with intimate interlayer contact; a discontinuous thin Al-rich oxide film is also evident at the coating–substrate interface, likely originating from corundum shot-blast preparation and in-flight oxidation. Optical and cross-section analyses indicate a coating thickness of 127.44 ± 5.27 µm and very low apparent porosity (image analysis average 0.71 vol.%). Vickers microhardness (HV0.5, ASTM E92) is relatively homogeneous through the layer with an average 317 HV0.5 (std. dev. 23); substrate bulk hardness averages 176 HV0.5 (std. dev. 23). Chemical etching and microstructural inspection show no detectable substrate microstructural alteration near the interface (no grain growth or recrystallization), indicating limited thermal impact on the substrate despite effective bonding and high coating density.

After exposure to molten lead and subsequent cleaning to remove lead, top-view SEM and EDX confirm (Figure 9) retention of the powder-derived surface morphology but a thicker, more homogeneous oxide scale, indicating growth of a protective alumina-rich barrier that limits Pb ingress. Cross-sectional inspection shows the coating remaining coherent with no observable lead penetration, corrosion channels, or interfacial degradation. Elemental mapping across the coating/substrate interface shows a localized Ni enrichment in the near-interface region, adjacent to the 316Ti-type substrate (Figure 9(IIc)). This feature may be associated with limited Ni redistribution from the substrate toward the coating during deposition and/or exposure. The local co-presence of Ni and Al suggests the possible formation of Ni–Al-rich reaction products in a narrow interfacial region; however, their exact nature cannot be confirmed by SEM-EDS alone and would require dedicated phase analysis.

### 3.8. Comparative Overview of Coating Systems

To facilitate direct comparison across the investigated material–technology combinations, Table 3 and Table 4 summarize the main as-deposited microstructural and mechanical characteristics and the corresponding post-exposure response, respectively.

## 4. Discussion

The horizontal screening presented here demonstrates that multiple coating chemistries and deposition technologies can perform well when exposed to liquid lead, provided their structural integrity remains intact. Under the tested conditions (e.g., 1000 h in stagnant lead at 600 °C with an oxygen concentration of 10^−7^ wt.%), the majority of the investigated systems successfully acted as an effective physical barrier, preventing corrosive attack on the underlying steel substrates, but a critical comparison of the pre-corrosion microstructural and mechanical metrics reveals important differences that govern robustness, likely failure modes, and component suitability. Coating thickness was not normalized across the investigated systems, as it is therefore treated as one of the process-dependent characteristics of each coating system.

Thermal spray methods (APS and HVOF) both produce lamellar, splat-type microstructures typical of high-temperature deposition, but they differ noticeably in defect density and the degree of oxidation: APS AFA and AFF deposits show measurable inter splat oxidation and microporosity (AFA porosity 1.98–3.13 vol.%, AFF up to 4.06 vol.%) and a shallow thermally-affected zone (25 µm) with localized recrystallization beneath the coating. HVOF yields denser, more consolidated layers with markedly lower apparent porosity (AFA 0.65 vol.%, AFF 0.71 vol.%), higher and more uniform hardness, and no detectable substrate recrystallization. These differences reflect the higher in-flight particle temperatures and oxidation kinetics in APS and the higher kinetic energy/lower oxidation balance achievable in HVOF, and they translate into distinct microstructural reservoirs for potential degradation. APS oxide-rich interlayers and lamellae increase the population of weak interfaces that could act as preferential paths for aggressive species, while HVOF’s compact microstructure presents a more uniformly dense barrier.

Cold spray stands apart as a solid-state route: optimized CGS AFA and AFF coatings are dense and dominated by heavily deformed particle imprints, with very low porosity (AFF < 0.9 vol.%, AFA < 2.4 vol.%) and elevated coating hardness (AFF 316 HV0.5, AFA 377 HV0.5). Critically, CGS introduces negligible thermal input to the substrate, producing no TAZ/HAZ or recrystallization and preserving the base-metal microstructure. This preservation of substrate condition, combined with low defect connectivity in the deposit, is a decisive advantage for applications where substrate cold-worked microstructure or irradiation-sensitive microstructure must be retained.

Electrodeposited chromium, while chemically pure and hard, exhibits a dense network of microcracks whose connectivity increases with thickness; this pre-existing crack population dominates its short-term performance and renders it highly susceptible to rapid lead infiltration, as reflected in post-test observations.

Across the Al-rich alloys (AFA and AFF), the ability to form Al-rich oxide scales during exposure provides a common chemical mechanism for limiting Pb ingress, but the deposition route strongly modulates how effectively that mechanism can operate. Where coatings are dense and defect connectivity is low (HVOF and optimized cold spray), barrier formation is effective, and substrate protection is consistent; where the deposition produces lamellar oxide inclusions, higher local porosity or interfacial oxides (APS), protection can still emerge through the growth of protective oxides, albeit with a higher risk of localized failures.

The localized Ni, Mn and Cr redistributions observed at selected coating/substrate and coating/Pb interfaces may result from thermally activated interdiffusion, local chemical-potential gradients and selective oxidation/reaction processes. In particular, Ni enrichment in Al-rich regions may indicate the formation of Ni–Al-rich reaction products. However, the present SEM-EDS observations do not allow a unique mechanistic assignment, and dedicated compositional profiling and phase identification would be required to distinguish among the possible diffusion and reaction pathways.

Equally important, process thermal management, not chemistry alone, is often the primary determinant of short-term reliability: APS-induced substrate modification (TAZ/recrystallization) may be unacceptable for core, cold-worked components even if the coating chemistry is protective, while cold spray’s low thermal footprint preserves substrate integrity.

The primary exception encountered in this study is the electrodeposited chromium system. While pure chromium is often cited in the literature as a promising coating material, the investigated Cr coatings exhibited a dense pre-existing microcrack network that provided preferential pathways for molten Pb infiltration. Pb access through these defects was associated with severe substrate degradation and significant Ni depletion, with a maximum corrosion depth of approximately 280 µm. The poor performance observed under the investigated conditions should therefore be associated primarily with the integrity and connectivity of the crack network rather than with Cr chemistry alone. In principle, optimization of the electrodeposition parameters or suitable post-deposition treatments aimed at reducing crack density and connectivity could improve barrier integrity. However, these strategies were not investigated in the present work, and their effectiveness cannot be assessed from the available results. Accordingly, the present findings should be restricted to the electrodeposited Cr coatings and processing conditions investigated in this study.

Beyond the corrosion response observed in liquid lead, the practical implementation of each coating route must be evaluated against the requirements of the target component. Substrate metallurgical state, component geometry, surface accessibility, expected loading conditions and service environment can strongly constrain the selection of suitable material–technology combinations.

For core components, such as fuel cladding, spacers and wrappers, particular attention must be paid to the preservation of the cold-worked microstructure of austenitic alloys. Their creep strength and void-swelling resistance depend on the retained dislocation substructure, which can be partly lost through recovery or recrystallization if the substrate is exposed to sufficiently high temperatures or prolonged thermal treatments [42]. In this respect, among the technologies investigated in this work, cold spray may be seen as the best match for thin-walled cold-worked substrates, owing to its solid-state nature, low thermal input, limited porosity and good coating cohesion. APS and HVOF remain technically viable coating routes, but their line-of-sight nature, higher thermal input and possible presence of pores, oxide inclusions or thermally affected regions make them less favorable for fatigue-sensitive, thin-walled core geometries.

Out-of-core sub-components, including pump shafts, impellers, valve seats and steam-generator tubes, are generally less constrained by irradiation damage and cold-work retention. However, they often involve more complex geometries and may be exposed to the combined effect of corrosion, erosion and wear loading or flow-induced vibration. For accessible line-of-sight surfaces, thicker APS or HVOF AFA/AFF coatings may therefore represent practical options, particularly where robust metallic barriers are required. Conversely, non-line-of-sight internal geometries, such as portions of steam-generator tube bundles, are not fully covered by the technology set considered in this paper and require dedicated work.

Chromium electroplating may retain its usefulness for components where tribological performance is also relevant, provided that coating cracking and lead-ingress pathways were adequately controlled [37].

For large structural components, such as the reactor vessel, the availability of large and relatively accessible surfaces makes robotically applied APS or HVOF coatings a potentially practical near-term solution. Nevertheless, these routes should be compared with weld overlay and other metallurgical routes [17], which may offer stronger bonding and reduced line-of-sight constraints. These alternative technologies fall outside the scope of the present screening and will be assessed in a subsequent publication.

Overall, the results support a differentiated selection strategy rather than a single coating solution for all LFR components. Cold spray is favored for thermally sensitive cold-worked substrates, HVOF appears promising for dense coatings on accessible surfaces, APS may be considered where deposition rate or specific implementation constraints are dominant, and Cr electroplating may be relevant only for selected applications requiring additional tribological functionality.

Finally, while the present 1000 h static Pb tests provide a consistent baseline, longer exposures, flowing lead conditions, mechanical loading, and irradiation-coupled experiments are needed to validate long-term reliability and to quantify failure probabilities for the systems investigated. Observations such as Ni migration at some interfaces and localized Mn/Cr enrichments warrant targeted studies on diffusion and reaction product formation to assess their implications for coating adhesion and barrier stability. Overall, this study indicates that deposition-induced defect populations and thermal aspects are as critical as alloy chemistry for short-term lead compatibility. Subsequent qualification efforts should prioritize process optimization and longer-term testing on cold spray and HVOF AFA/AFF systems.

## 5. Conclusions

This horizontal screening provides a comparative assessment of candidate coating systems for the protection of structural materials exposed to liquid lead under controlled conditions. The results show that several Al-rich metallic coatings deposited by APS, HVOF and CGS can effectively limit substrate corrosion after exposure to stagnant liquid lead for 1000 h at 600 °C and 10^−7^ wt.% oxygen. In contrast, the electrodeposited Cr coatings investigated in this study showed severe degradation, mainly associated with the pre-existing microcrack network and subsequent Pb ingress, and are therefore not considered suitable in their present form for liquid-lead corrosion protection.

This study confirms that coating performance cannot be assessed based on chemistry alone. Deposition route, coating microstructure, defect population, coating continuity and interface quality play a critical role in determining the protective behavior of the investigated systems. Future down-selection must also consider the compatibility between coating technology and target component, including substrate metallurgical state, component geometry, surface accessibility and expected service conditions.

Within the scope of this first screening and under the investigated static liquid-Pb exposure conditions, cold spray, HVOF and APS of Al-rich AFA and FeCrAl-type alloys emerge as the most promising candidates for further qualification among those investigated, although their applicability will depend on the specific component class and qualification requirements.

These findings provide a first experimental basis for progressing from chemical compatibility screening toward application-oriented coating selection.

Future work will extend the assessment to additional material–technology combinations, longer exposure times, different oxygen concentrations, flowing lead, mechanical loading and irradiation-relevant conditions, with the aim of supporting component-specific qualification and, ultimately, standardization within the relevant nuclear design-code framework.

## Figures and Tables

**Table 1:** Nominal chemical composition of the proprietary AFA steel powder used for APS deposition.

Element	Fe	Ni	Cr	Mn	Al	Nb	Ti	Si	C	O	N	S
Comp.wt.%	Bal.	13.45	11.75	7.4	2.7	0.36	0.23	0.26	0.12	0.025	0.003	0.003

**Table 2:** Nominal chemical composition of the proprietary AFA steel powder used for CGS deposition.

Element	Fe	Ni	Cr	Mn	Al	Nb	Ti	Si	C	O	N	S
Comp.wt.%	Bal.	13.5	11.96	7.4	2.65	0.38	0.24	0.26	0.12	0.03	0.003	0.003

**Table 3:** As-deposited coating characteristics.

Material	Technology	Thickness (µm)	Porosity † (vol.%)	Hardness	Main Defects §	Interface/TAZ §
**AFA**	APS	142.1 ± 3.5	1.98–3.13 (avg. 2.64)	254 ± 48.4 HV0.1	Lamellar splats; microporosity; cracks; interparticle oxides	TAZ ≈ 25 µm; localized recrystallization
**AFF**	APS	99.50 ± 3.93	0.82–4.06	236 ± 20.7 HV0.1	Microporosity; interlayer oxidation	TAZ ≈ 25 µm; localized recrystallization
**AFA**	CGS	60 ± 4	<2.4	377 ± 15.0 HV0.5	Dense structure; occasional pores	No TAZ detected
**AFF**	CGS	77 ± 5	<0.9	316 ± 21.8 HV0.5	Dense structure; occasional interface voids	No TAZ detected
**Cr**	Electroplating	4.6/24.2/36.5/118 *	n.r.	720/868/613/920 HV *	Dense microcrack network; connectivity increases with thickness	n.r.; deposition < 60 °C
**AFA**	HVOF	93.4 ± 3.9	0.65	326 ± 19.3 HV0.5	Limited porosity; occasional microcracks	No substrate alteration detected
**AFF**	HVOF	127.44 ± 5.27	0.71	317 ± 23 HV0.5	Dense splat structure	No substrate alteration; thin interfacial Al-rich oxide

Notes: **†** Porosity values are obtained from threshold-based analysis of selected two-dimensional cross-sections and are intended as semi-quantitative descriptors of coating compactness and defect population. Because the number of analyzed fields, pore morphology, orientation, and segmentation response differ among coating technologies, these values should not be interpreted as statistically equivalent measurements or used as a stand-alone quantitative ranking parameter. **§** Defect and interface descriptions are based on metallographic/SEM observations and are qualitative unless a specific dimension is reported. * Cr hardness values correspond respectively to the listed coating thicknesses; nanoindentation-to-Vickers conversion was required for the thinner deposits.

**Table 4:** Post-exposure behavior after 1000 h in liquid Pbm (600 °C 10^−7^ wt.% O_2_).

Material	Technology	Max Pb Penetration in Coating (µm)	Max Substrate Corrosion (µm)	Main Observations	Final Condition
**AFA**	APS	≈62.9	Not observed	Local Pb interaction; Mn enrichment; uncoated area corroded	Protective, locally affected
**AFF**	APS	Not observed	Not observed	Protective oxide regions; no through-coating pathways	Protective
**AFA**	CGS	n.r.	Not observed	Al–O surface enrichment; O/Cr/Mn at interface	Protective
**AFF**	CGS	n.r.	Not observed	Al-rich oxide; localized Ni redistribution	Protective
**Cr**	Electroplating	n.a.	≈280	Coating loss; microcrack-assisted Pb ingress; Ni depletion	Severe degradation
**AFA**	HVOF	n.r.	Not observed	Al/Mn-rich protective surface scale	Protective
**AFF**	HVOF	Not observed	Not observed	No Pb ingress or corrosion channels; Al-rich oxide	Fully protective

## Data Availability

The original contributions presented in this study are included in the article. Further inquiries can be directed to the corresponding author.

## Abbreviations

The following abbreviations are used in this manuscript:

APS	Atmospheric plasma spraying
HVOF	High-velocity oxy-fuel spraying
AFA	Alumina-forming austenitic steel
AFF	Alumina-forming ferritic steel
TAZ	Thermally affected zone
CGS	Cold gas spraying
